# Pathogenesis and Treatment of Myeloma-Related Bone Disease

**DOI:** 10.3390/ijms23063112

**Published:** 2022-03-14

**Authors:** Yuh-Ching Gau, Tsung-Jang Yeh, Chin-Mu Hsu, Samuel Yien Hsiao, Hui-Hua Hsiao

**Affiliations:** 1 Division of Hematology and Oncology, Department of Internal Medicine, Kaohsiung Medical University Hospital, Kaohsiung 80756, Taiwan; cheesecaketwin@gmail.com (Y.-C.G.); aw7719@gmail.com (T.-J.Y.); e12013@gmail.com (C.-M.H.); 2 Graduate Institute of Clinical Medicine, College of Medicine, Kaohsiung Medical University, Kaohsiung 80708, Taiwan; 3 Department of Biology, University of Rutgers-Camden, Camden, NJ 08102, USA; ucdsacnyu@gmail.com; 4 Cancer Center, Kaohsiung Medical University Hospital, Kaohsiung 80756, Taiwan; 5 Center for Cancer Research, Kaohsiung Medical University, Kaohsiung 80708, Taiwan; 6 Faculty of Medicine, Kaohsiung Medical University, Kaohsiung 80708, Taiwan

**Keywords:** myeloma, myeloma bone disease, osteoclastogenesis, bisphosphonates, denosumab, novel agents

## Abstract

Multiple myeloma is a hematologic malignancy of plasma cells that causes bone-destructive lesions and associated skeletal-related events (SREs). The pathogenesis of myeloma-related bone disease (MBD) is the imbalance of the bone-remodeling process, which results from osteoclast activation, osteoblast suppression, and the immunosuppressed bone marrow microenvironment. Many important signaling cascades, including the RANKL/RANK/OPG axis, Notch signaling, the Wnt/β-Catenin signaling pathways, and signaling molecules, such as DKK-1, sclerostin, osteopontin, activin A, chemokines, and interleukins are involved and play critical roles in MBD. Currently, bisphosphonate and denosumab are the gold standard for MBD prevention and treatment. As the molecular mechanisms of MBD become increasingly well understood, novel agents are being thoroughly explored in both preclinical and clinical settings. Herein, we will provide an updated overview of the pathogenesis of MBD, summarize the clinical management and guidelines, and discuss novel bone-modifying therapies for further management of MBD.

## 1. Introduction

Multiple myeloma (MM), also known as plasma cell myeloma, is the second-most common hematological malignancy and is characterized by the malignant proliferation of monoclonal plasma cells in the bone marrow, leading to bone destruction, marrow failure, and associated end organ damage (hypercalcemia, renal insufficiency, anemia, or bone lesions) [1]. According to statistics for the United States, MM accounts for about 1.8% of all cancers and 18% of hematologic malignancies. MM is most frequently diagnosed in people aged 65 to 74 years; the median age is 69 years [2]. The incidence of MM is increasing worldwide, but the rate of increase is greatest in Asia. In Taiwan, the age-adjusted incidence of MM increased by 13% between 2007 and 2012 [3]. The overall survival of patients with MM has improved significantly in the last two decades because of novel therapeutic agents, but it remains an incurable disease in the majority of patients. For newly diagnosed patients, the median overall survival is approximately six years [4].

MM-related bone disease (MBD) is a hallmark of MM. More than 80% of MM patients develop osteolytic bone lesions at diagnosis [5]; these patients are at high risk of skeletal-related events (SREs), including pathological fractures, spinal cord compression, and the need for surgical or radiotherapeutic intervention [6]. Up to 60% of MM patients will develop pathologic fracture during the disease course [7]. SREs have a negative impact on patient survival, quality of life, and public health costs [6].

The pathogenesis of MBD is the imbalance and uncoupling of the bone-remodeling process, due to the dysregulation of the bone marrow microenvironment [8,9], increased osteoclast formation and activity, and osteoblast suppression [10]. Both the direct and indirect interactions between MM cells and osteocytes, osteoclasts, osteoblasts, immune cells, and bone mesenchymal stem cells (BMSCs) participate in the complex pathogenesis event.

Regarding the MBD therapeutics, the current guidelines suggests that bisphosphonates (namely zoledronic acid or pamidronic acid) should be administered to all patients with active MM, regardless of the presence of MBD in imaging study; denosumab, a humanized monoclonal antibody directed against RANKL, is used for patients with MBD on imaging or for patients with renal impairment [11]. In addition, novel agents have been developed from the understanding of the molecular pathways of MBD pathogenesis.

Here, we review the pathogenesis, treatment modalities, and preclinical research and clinical trials for investigating novel bone-modifying agents for MBD management.

## 2. Increased Bone Resorption by Osteoclasts

### 2.1. RANKL/RANK/OPG Axis

The pathogenesis of MBD is primarily in the upregulation of osteoclast differentiation and activity resulting in unbalanced bone resorption, which causes characteristic osteolytic lesions [12]. The two main factors required for osteoclast differentiation are macrophage-colony stimulating factor (M-CSF) and receptor activator of NFκB (RANK) ligand (RANKL) [13,14]. The key pathway, receptor activator of nuclear factor-κB ligand (RANKL)/RANK/osteoprotegerin (OPG), regulates the formation of multinucleated osteoclasts from their precursors, as well as their activation and survival in normal bone remodeling [15,16]. Osteocytes produce the majority of RANKL, but activated lymphocytes, BMSCs, and endothelial cells also produce RANKL. RANKL promotes osteoclast activity by binding to RANK on the membrane of osteoclastic lineage cells [15]. OPG, a soluble decoy receptor and a member of the TNF receptor family [17], is secreted by osteoblasts, BMSCs, and osteocytes; it protects bone from excessive resorption by binding to RANKL and preventing it from binding to RANK [15].

MM cells degrade OPG through the membrane syndecan (CD138)-1 system [18]. The RANKL/OPG ratio is a critical regulator of the bone resorption rate; increased RANKL/OPG is found in the MM microenvironment and serum RANKL/OPG is negatively correlated with patient survival [15,19,20]. Increased osteoclast activity, induced by osteoclast-derived osteopontin and vascular endothelial growth factor (VEGF) from myeloma cells, appears to contribute both to the increased angiogenesis and tumor growth in MM [18,21].

### 2.2. Notch Signaling Pathway

The Notch signaling pathway is also implicated in MM-induced osteoclastogenesis [22,23]. Four transmembrane receptors (Notch 1–4) are included in the Notch family, and they bind to their ligands (Jagged 1,2 and Delta-like 1,3,4) expressed by neighboring cells. The Notch family, expressed in the membranes of MM cells, and its activation by homotypic or heterotypic interactions and the subsequent intracellular cascade are important for the differentiation of pre-osteoclasts, which trigger the secretion of angiogenic factors by BMSCs [22]. The inhibition of Notch pathway causes decreased MM cell proliferation, induces MM cell apoptosis, and inhibits osteoclastogenesis [24]. The systemic inhibition of Notch with γ-secretase inhibitors (GSIs) decreases multiple myeloma tumor growth. A novel agent forbone-targeted Notch inhibition (BT-GSI) has both anti-myeloma and anti-resorptive properties, and is under investigation [25].

### 2.3. Chemokines: CCL-3 (MIP-1α)/CCR1, CCR5

Chemokine (C-C motif) ligand 3 (CCL-3), also called macrophage inflammatory protein-1α (MIP-1α), is a chemokine with inflammatory and chemokinetic properties secreted by MM plasma cells that plays a critical role in the pathogenesis of MBD [26]. The binding of CCL-3 to its G-protein coupled receptors, CCR1 and CCR5, activates the ERK and AKT signaling pathways and contributes to the development of bone disease in MM by supporting tumor growth and regulating osteoclast differentiation [26,27]. In MM human cell lines with translocation t(4;14), the overexpression of fibroblast growth factor receptor 3 (FGFR3) is induced by MIP-1α promoter activity, as well as the MIP-1α gene and protein expression/secretion [28]. CCL3 induces osteoclast formation in a RANK/RANKL-dependent manner both in vivo and in vitro studies [29,30]. A higher level of CCL-3 in the bone marrow is positively associated with the increased presence of osteolytic bone disease [31]. On the other hand, CCL3 also inhibits osteoblastogenesis through the Runx2/osterix pathway and causes the suppression of mineralization activation and osteocalcin expression [32]. Treatment with the CCR1-specific antagonist MLN3897 inhibits the interaction of multiple myeloma cells and osteoclasts and impedes osteoclastogenesis in vitro [33].

### 2.4. Chemokines: CCL-20(MIP-3α)/CCR6

Chemokine (C-C motif) ligand 20 (CCL-20), also known as macrophage inflammatory protein-3α (MIP-3α), and its receptor CCR6 are upregulated in the bone microenvironment by MM cells and contribute to osteoclast formation and osteolytic bone lesions in MM patients [34]. The CCL-20 level in the bone marrow has a significant predictive value for osteolytic bone lesions [31].

### 2.5. BTK and CXCL-12 (SDF-1)/CXCR4

Bruton’s tyrosine kinase (BTK) is a nonreceptor tyrosine kinase of the TEC family and plays a crucial role in oncogenic signaling that is critical for the proliferation and survival of leukemic cells in many B cell malignancies and osteoclast differentiation [35,36]. CXCL-12 [stromal cell-derived factor-1 (SDF-1)] is a homeostatic chemokine that binds primarily to the CXC receptor 4 (CXCR4; CD184) [37]. After CXCL-12 and CXCR4 binding, the homing of myeloma cells and osteoclastogenesis are triggered [38]. BTK is found to be expressed in MM cells, and has a positive correlation with CXCR4 expression. In an in vitro study, BTK inhibition reduced the migration of myeloma cells toward SDF-1 [39]. The interesting role of BTK activity in myeloma cell clonogenicity and metastasis and in osteoclast-mediated bone resorption may have therapeutic potential in MBD. The BTK inhibitor ibrutinib is now in use in combination with other anti-myeloma agents in relapse and refractory clinical trial settings.

### 2.6. Annexin II (AnxA2, A2)

Annexin II (AnxA2, A2) is a Ca^2+^-dependent, anionic phospholipid-binding protein that belongs to the ubiquitous multigene annexin family, expressed on most of the endothelial cells, mononuclear macrophage, marrow cells, and some tumor cells [40]. Preclinical studies show that AnxA2 is highly expressed in myeloma cells from MM patients and can promote myeloma cell growth, reduce apoptosis in myeloma cell lines, and increase osteoclast formation [41,42]. Higher AnxA2 expression in myeloma cells is associated with significantly more adverse prognostic features, and inferior event-free and overall survival [42].

### 2.7. Osteopontin (OPN)

Osteopontin (OPN) is a non-collagenous matrix protein produced by a variety of cells, including osteoblasts, osteoclasts, and several types of tumor cells [43]. OPN is associated with inflammation and tissue remodeling [44]. Osteoclast-derived OPN and VEGF from myeloma cells cooperatively enhance angiogenesis and induce osteoclastogenic activity by vascular endothelial cells [45]. High OPN expression correlates with higher tumor burden, and greater bone destruction [46]; OPN may also play a critical role in MM progression and osteoclastogenesis [47].

### 2.8. Interleukins (IL-3, IL-6, IL-17)

#### 2.8.1. Interleukin 3 (IL-3)

Interleukin 3 (IL-3) acts as a bifunctional cytokine that indirectly increases osteoclastogenesis and suppresses osteoblastogenesis in MM cells in vitro [48]. The IL-3 level in bone marrow serum significantly elevated in MM patients. IL-3 can induce osteoclastogenesis in human bone marrow cultures, which was inhibited by a blocking antibody to IL-3 [49]. In addition, IL-3 influences indirectly the growth of osteoclasts by inducing activin A production [48] and increasing RANKL and MIP-1α [49].

#### 2.8.2. Interleukin 6 (IL-6)

Interleukin 6 (IL-6), an inflammatory cytokines, can modulate skeletal homeostasis and osteoclast differentiation [50]. IL-6 stimulates osteoclast differentiation only when IL-6 binding with soluble IL-6 receptor (sIL-6R) via enhancing the expression of RANKL and OPG, but decreasing RANK expression [51]. In addition, IL-6 enhances bone resorption by promoting the proliferation of Dickkopf-1 (DKK-1)-secreting myeloma cells, but DKK-1 secretion is blocked after IL-6 neutralizing agents [52].

#### 2.8.3. Interleukin 17 (IL-17)

Interleukin-17 (IL-17) and IL-17-producing cells (T-helper cells, Th17) play important roles in inflammation and the immune response [53]. Research conducted in MM cell models shows that IL-17–producing T cells induce osteoclast activation and that IL-17 production directly correlates with lytic bone disease, irrespective of the tumor burden, indicating that the Th17 T-cell phenotype is a key predictor of lytic bone disease in MM [54].

### 2.9. TGFβ Superfamily and Activin-A

Activin-A is a dimeric multifunctional glycoprotein that belong to the transforming growth factor-β (TGF-β) superfamily and regulates a broad spectrum of biological functions, including bone remodeling [55]. Activin-A has growth stimulatory effects on osteoclasts by inducing RANK expression and activating the NF-κB pathway [56], and medicates osteoblast function inhibition [57,58]. Increased bone marrow plasma activin A levels are associated with MM patients developing extensive osteolytic disease [57]. In addition, higher circulating Activin-A in myeloma patients is correlated with more advanced disease and poorer prognosis [59]. In preclinical studies in mouse models, the administration of anti-Activin-A agents, the Activin-A chimeric inhibitor (RAP-011) derived from the fusion of the extracellular domain of activin receptor IIA and the constant domain of the murine IgG2a or a soluble Activin-A receptor type IIA fusion protein (ActRIIA.muFc) successfully inhibits osteolytic bone lesions developing by both inhibiting osteoclastogenesis and stimulating osteoblastogenesis [60]. A Phase II clinical trial investigating MM patients with osteolytic lesions treated with Sotatercept (ACE-011) combined with other anti-MM agents revealed that it was safe and well tolerated [61].

### 2.10. TNF (Tumor Necrosis Factor) Superfamily

TNF-α is one of the most potent osteoclastogenic cytokines produced in inflammation, and directly targets macrophages within a stromal environment that expresses permissive levels of RANKL [62]. B-cell-activating factor of the TNF family (BAFF; also known as B lymphocyte stimulator and TNFSF-13B), is secreted and expressed predominantly by macrophages, dendritic cells, osteoclasts, and BMSCs, and provides a key survival signal for the maturation of peripheral B cells that play a regulatory role in osteoblast differentiation [63,64]. The ligation of BAFF to its receptor can cause constitutive activation of either the canonical or non-canonical NF-κB pathways, resulting in the upregulation of anti-apoptotic proteins and the downregulation of pro-apoptotic proteins [64,65], enhanced MM cell survival, and MBD progression [66,67]. A Phase II clinical trial of the anti-BAFF monoclonal antibody, tabalumab, used in previously treated MM, did not show PFS benefit compared to placebo [68].

## 3. Suppression of Bone Formation by Osteoblasts

### 3.1. Wnt/β-Catenin Signaling Pathway

The Wnt signaling is very important in skeletogenesis as it promotes the proliferation, expansion, and survival of immature osteoblastic cells [69]; it also plays an important role in MBD pathogenesis [70]. The formation of the Wnt-Frizzled-low-density lipoprotein-related protein (LRP) complex activates Wnt/β-catenin signaling in the canonical pathway by the activation of Disheveled (Dvl), which inhibits glycogen synthase kinase 3β (GSK3β) from phosphorylating β-catenin. The cytoplasmic level of β-catenin consequently rises, and β-catenin translocates into the nucleus to bind with the transcriptional factor T-cell factor (Tcf)/lymphoid enhancer-binding factor (Lef-1), upregulating the expression of target genes such as cyclin D1, axin2, c-Myc, and peroxisome proliferator-activated receptor (PPARδ), causing bone formation, and impeding bone resorption [71,72]. On the other hand, the existence of Wnt antagonists, Dickkopfs-1 (DKK-1), sclerostin, and secreted frizzled-related proteins (sFRPs), impairs osteoblastogenesis and blocks bone formation by impeding the Wnt signaling cascade [69,72,73]. In MM, soluble canonical Wnt inhibitors produced from MM cells and BMSCs that interrupt Wnt signaling are increased, which causes severe osteoblast/osteoclast imbalance via the upregulation of the RANKL/OPG ratio [74,75,76].

### 3.2. DKK-1, Sclerostin

Dickkopf-1 (DKK-1) is a secreted protein, a member of the DKK family, and is important in vertebrate development, including osteoblastogenesis [76]. DKK1 is secreted by MM cells and BMSCs; it blocks the maturation of osteoblasts and the formation of mineralized matrix by antagonizing the canonical Wnt pathway through binding to LRP5/6 [70,71,72,74,75,76]. In addition, DKK-1 prevents the differentiation of MSCs into osteoblasts, and the undifferentiated MSCs produce IL-6, which stimulates the proliferation of DKK1-secreting MM cells. This vicious cycle continues, resulting in more extensive osteolytic lesions [52]. In MM, BMSCs produce increased amounts of DKK-1 [77]. DKK-1 serum and bone marrow plasma concentrations correlate with the extent of MBD [78]. Anti-DKK-1 strategies are valuable since high serum levels of DKK1 are correlated with osteolytic lesion formation. A variety of DKK-1 antibodies has emerged; most have shown encouraging results in MM cell lines in both preclinical and clinical trials [76]. The DKK1-neutralizing antibody BHQ880 upregulates β-catenin levels, downregulates NF-κB activity, increases osteoblast differentiation, neutralizes the negative effect of osteoblastogenesis, and reduces IL-6 secretion [70,79]. In a Phase II trial, BHQ880 administered as a monotherapy was well tolerated in previously untreated high- and intermediate-risk SMM patients with increased anabolic bone activity [80]. The DKK1-DNA vaccine can be used for immunotherapy of patients with MM, and was effective in reducing tumor burden in mice with established MM in a preclinical study [81].

Sclerostin is a small glycoprotein expressed by the SOST gene, and is secreted by osteocytes during bone remodeling [82]. During bone formation, sclerostin binds to LRP5/6 to inhibit the Wnt signaling pathway, completing a negative feedback loop of osteogenesis [82,83]. Sclerostin competed with the type I and type II bone morphogenetic protein (BMP) receptors for binding to BMPs, de-regulated BMP signaling, and suppressed the mineralization of osteoblastic cells [84]. Sclerostin, also secreted by MM cells, mediates the upregulation of RANKL and the downregulation of OPG in osteoblasts and contributes to the suppression of bone formation in the MBD [73,85]. Dkk-1 and sclerostin have synergic effects, resulting in osteoblast dysfunction [72]. Many clinical studies have shown the positive correlation the levels of circulating sclerostin and the presence of osteolytic fractures, disease stage, and biochemical markers of bone remodeling in MM patients [86,87,88]. Anti-sclerostin antibodies (Scl-Ab), such as romosozumab and blosozumab, have been tested in osteoporosis treatment, revealing potent activity in stimulating bone formation and reducing bone resorption [89,90]. The combination of Scl-Ab and anti-myeloma agents, or the osteoclast inhibitor zoledronic acid, has been investigated in preclinical studies [91,92,93].

### 3.3. Runt-Related Transcription Factor 2 (RUNX2)

Runt-related transcription factor 2 (RUNX2) belongs to the family of runt-related transcription factors, and plays an essential role in both osteoblast differentiation and the expression of osteoblast-specific genes [94]. RUNX2/CBFA1 (core-binding factor Runt domain alpha subunit 1) is a critical osteoblast transcription factor in the inhibition of osteoblastogenesis in MM [95]. Increased Runx2 expression is significantly associated with a high-risk myeloma population, and promotes MBD progression [96].

### 3.4. EphrinB2/EphB4 Signaling Pathway

Together, Eph tyrosine kinase receptors and their ephrin ligands form a critical cell communication system in normal physiology and disease pathogenesis [97]. The bidirectional signaling between the cell-surface ligand EphrinB2 (expressed in osteoblasts) and its receptor, EphB4 (expressed in osteoblasts and BMSCs), is involved in the coupling of osteoblastogenesis and osteoclastogenesis and in angiogenesis [98,99]. EphrinB2/EphB4 signaling links the suppression of osteoclast differentiation by suppressing Fos and Nfatc1 transcription to the stimulation of bone formation by RhoA downregulation [99]. In MM patients, both EphrinB2 and EphB4 expression is decreased in BMSCs. In murine MM models, the use of EphrinB2-Fc and EphB4-Fcs can enhance bone formation, inhibit myeloma growth, and reverse the pathogenesis of MBD.

## 4. Current Myeloma-Related Bone Disease Treatment

### 4.1. Bisphosphonates

Bisphosphonates (BPs) are the cornerstone for MBD treatment because they can prevent, reduce, and delay MM-related skeletal complications [11,100,101]. During bone remodeling, bisphosphonates act as pyrophosphate analogs that bind to exposed areas of hydroxyapatite crystals, and osteoclasts endocytose bisphosphonates, which are potent inhibitors of intracellular farnesyl pyrophosphate synthase, impairing osteoclastogenesis and enhancing osteoblostogenesis [102]. The FDA-approved BPs for MBD are zoledronic acid and pamidronate [103]. Clodronate (oral non-nitrogenous BP), which is used for reducing SREs in MM patients, yielded inferior survival outcomes compared with zoledronic acid in the Myeloma IX trial [104,105,106]. Zolendronic acid may have an anti-myeloma effect through the inhibition of protein prenylation and the inhibition of antiangiogensis or by the indirect downregulation of BMSC-related adhesion molecules and the blocking of osteoclast activation [8,107,108]. According to the long-term follow-up data in one large Phase III trial, zoledronic acid was more effective than pamidronate in MM patients with bone metastases. The advantages of zoledronic acid (4 mg) include lower mean skeletal morbidity rate, increased median time to first SRE, and reduced risk of developing an SRE compared with pamidronate (90 mg) [109]. BPs are recommended as standard treatment of MM patients with either osteolytic bone lesions or osteopenia in many current guidelines, including those from the National Comprehensive Cancer Network (NCCN) [110], the American Society of Clinical Oncology (ASCO) [111], the Mayo Clinic [112], the European Society for Medical Oncology (ESMO) [113], and the International Myeloma Working Group (IMWG) [11]. Zoledronic acid or pamidronate once monthly, at least for the first 1 to 2 years, is recommended for almost all patients with MM who have evidence of MBD [11,111,112,113] (Table 1). The standard dosing schedule for symptomatic MM patients with normal renal function is 4 mg intravascular infusion over 15 min every 3 to 4 weeks for zoledronic acid, and 30 mg or 90 mg administered over 45 min (for 30 mg) or 2 h (for 90 mg) every 3 to 4 weeks for pamidronic acid [11]. Dose adjustments for renal impairment are required both at diagnosis and during treatment. Zoledronic acid is not recommended for patients with severe renal impairment, while pamidronic acid can be administered increasing the time of administration to 4–6 h for them (whose creatinine clearance < 30 mL/min) [114]. Approximately 40% of patients treated with intravascular nitrogen-containing BPs may have a flu-like syndrome [114]. Some patients might develop severe hypocalcemia, so calcium and vitamin D supplements should be administered to all patients receiving BPs [11]. Some MM patients receiving BPs might have renal toxicity. Zoledronate has mainly been associated with acute tubular necrosis (ATN), and pamidronate causes collapsing focal segmental glomerulosclerosis (FSGS) and other patterns of glomerular disease [115]. Another well-known adverse effect of BPs is osteonecrosis of the jaw (ONJ), primarily associated with the long-term use of BPs, a tooth extraction or other surgical or invasive dental procedure, or a history of glucocorticoid use [11,114,116,117,118].

### 4.2. Denosumab

Denosumab is a fully human and highly specific monoclonal IgG2 antibody against RANKL, which inhibits the development and activity of osteoclasts, decreases bone resorption, and increases bone density [119]. Denosumab imitates the physiological effect of OPG by directly competing against the interaction of RANKL with RANK, and inhibits osteoclastogenesis [120]. Denosumab given 60 mg every 6 months subcutaneously was initial investigated for osteoporosis in postmenopausal osteoporosis and other metabolic bone diseases [119]; it successfully prevented fractures in postmenopausal women with osteoporosis [121], and had clinical benefit in patients with bone metastases from prostatic, breast, and lung malignancies [122]. In clinical trials, denosumab also shows clinical benefit in MM patients (Table 2) [123,124]. Denosumab 120 mg administered subcutaneously every 4 weeks was non-inferior to zoledronic acid 4 mg given as an intravenous infusion, delaying the time to first SRE after a multiple myeloma diagnosis in an international Phase III, randomized, double-blind 20090482 trial (Table 2) [125]. In the exploratory result of the 20090482 trial, the autologous stem cell transplantation (ASCT)-intent subgroup demonstrated the largest PFS benefit for denosumab compared with zoledronic acid [126]. Denosumab is also a hypercalcemia therapeutic strategy for MM patients [127]. For patients with renal dysfunction, denosumab is preferable over BPs, and denosumab can be safely administered with close monitoring of patients’ renal function [127,128,129]. As for safety issues, there appears to be slightly greater renal toxicity with zoledronic acid, but higher rates of hypocalcemia with denosumab [129]. Denosumab should be administered continuously until unacceptable toxicity occurs [11], because the rebound phenomenon may contribute to the resultant high soluble RANKL/OPG ratio, which is associated with an expanded pool of osteoclast precursors [130,131]. In contrast to BPs, denosumab does not incorporate into bone matrix; therefore, bone turnover is not suppressed after its cessation [130]. Denosumab discontinuation leads to reduced bone mineral density and increased risk of fracture [131]. The IMWG recommends that a single dose of zoledronic acid be given at least 6 months after the last dose of denozumab to prevent rebound effects [11].

## 5. Proteasome Inhibitors in Myeloma Bone Disease

Proteasome inhibition has emerged as an essential therapeutic strategy in the treatment of MM. Over the past decades, a variety of new proteasome inhibitors (PIs) have led to great progress in treatment and improved the survival of patients with MM [133]. Proteasome inhibition regulates bone metabolism through the reduction of RANKL-mediated osteoclast differentiation [134]. As mentioned above, RANKL binds to RANK on the surface of osteoclast precursors and subsequently causes NF-κB activation; therefore, PIs can block this pathway and inhibit osteoclastogenesis and suppress bone resorption [135,136]. In addition to the anti-myeloma effect, PIs have an anabolic effect on bone formation by inhibition of the ubiquitin–proteasome pathway [134]. PIs also induce the activation of Wnt/β-catenin signaling-independent Wnt ligands [134]. Bortezomib, a potent proteasome inhibitors, inhibits MM-BMSC interactions, and can activate β-catenin/TCF signaling in inducing osteoblast differentiation, and also upregulate RUNX-2 expression and enhance osteoblastogenesis [137,138]. Second-generation PIs, such as carfilzomib (PR-171), which selectively and irreversibly inhibits proteasome enzymatic activities in a dose-dependent manner, and ixazomib (MLN9708), which was the first oral PI with a robust efficacy and favorable safety profile, demonstrate clinical benefit in myeloma bone diseases through the inhibition of bone resorption and the promotion of osteoblastogenesis [133,139].

## 6. Supportive Intervention

Palliative radiotherapy also plays an important role for MM patients with MBD, as most have painful bone lesions. Radiotherapy is also used for the prophylactic treatment of impending pathological fractures, spinal cord compression, and the management of local neurological symptoms [140]. Surgical interventions with percutaneous vertebroplasty and balloon kyphoplasty are also applied to patients with vertebral compression fractures that have a poor response to conservation treatment [141,142].

## 7. Novel Therapeutic Agents in Preclinical Research and Ongoing Trials

**Figure 1:**
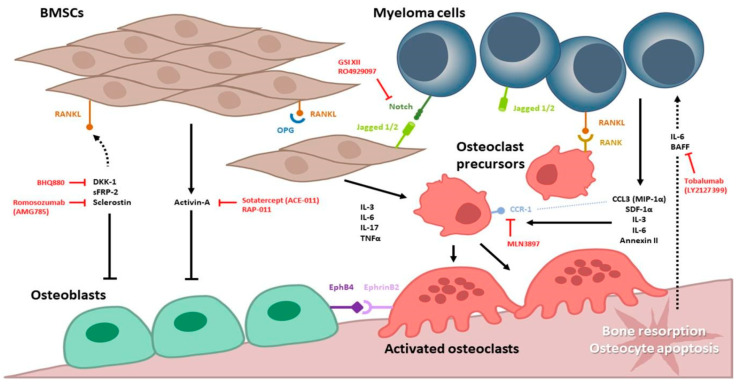
Schematic overview of myeloma bone disease and novel agents.

Wnt pathway signaling has a strong influence on MBD (Figure 1). The soluble Wnt inhibitor DKK-1, produced by MM cells, inhibits osteoblast activity, and its serum level correlates with focal bone lesions in MM [70]. BHQ880, a DKK1-neutralizing antibody, can increase osteoblast differentiation, neutralize the negative effect of MM cells on osteoblastogenesis, reduce IL-6 secretion, upregulate β-catenin levels, and downregulate nuclear factor-κB (NF-κB) activity in BMSCs in in vitro study [70,79]. In a Phase I/II study, BHQ880 and zoledronic acid in combination with anti-MM therapy were used in patients with relapsed or refractory MM with a prior SRE. The safety of BHQ880 was determined and BHQ880 results in a general trend towards increased bone mineral density over time [143,144] (Table 3). BHQ880 monotherapy in previously untreated patients with high- and intermediate-risk smoldering MM can cause anabolic bone activity, as shown using a novel imaging modality in one Phase II trial (NCT01302886) [80] (Table 3). Activin, which belongs to the TGF β superfamily, regulates bone remodeling and is involved in osteoclast development and differentiation. Sotatercept (formerly known as ACE-011), a recombinant activin receptor type IIA (ActRIIA) ligand trap comprising the extracellular domain of the human ActRIIA and human immunoglobulin G, has positive effects on bone metabolism and hematopoiesis in newly diagnosed and relapsed MM patients [61] (Table 3). RAP-011, a murine ortholog of sotatercept (Activin receptor type II Murine Fc Protein), combined with lenalidomide resulted in the effectively restoration of osteoblast function and inhibited MM-induced osteolysis in a preclinical setting [145] (Table 3).

B-cell activating factor (BAFF) is a member of the tumor necrosis factor superfamily (TNFSF). Tabalumab is a human IgG4 anti-BAFF monoclonal antibody [153]. Treatment of mice with tabalumab resulted in a significant reduction in tumor burden, prolonged survival, decreased osteoclast recruitment and activation, which caused fewer lytic lesions in the bone by a reduction in NF-κB signaling [154]. In Phase I studies, tabalumab in combination with bortezomib was well tolerated for patients with relapsed/refractory MM [147,148]. In a Phase II trial, patients with relapsed/refractory multiple myeloma were randomly assigned 1:1:1 to receive placebo, tabalumab 100 mg, or tabalumab 300 mg, each in combination with dexamethasone and bortezomib. There was no PFS benefit during treatment with tabalumab compared to placebo. A higher dose of 300 mg tabalumab did not improve efficacy compared to the 100 mg dose [68] (Table 3).

Bruton tyrosine kinase (BTK) inhibitors impaired osteoclastogenesis and suppressed bone resorption activity in an in vitro study [155]. Ibrutinib is a first-in-class, oral, covalent inhibitor of BTK that has produced notable responses in combination with dexamethasone in heavily pre-treated MM patients in a Phase II trial [149] (Table 3).

Anti-sclerostin antibody (Scl-Ab), such as romosozumab (AMG785), is well-studied in postmenopausal women with osteoporosis [89]. Sclerostin is a glycoprotein inhibitor of osteoblast Wnt signaling produced by osteocytes, which causes a decrease in bone formation [156]. Preclinical studies showed that treatment with anti-sclerostin antibody prevented myeloma-induced bone loss, reduced osteolytic bone lesions, and increased fracture resistance [91] (Table 3).

The γ-secretase inhibitor XII (GSI XII) impaired murine osteoclast differentiation in an in vitro study [151]. RO4929097 is a potent γ-secretase inhibitor (GSI), blocks Notch signaling, and reduces expression of the Notch transcriptional target gene, which is associated with osteoclastogenesis in MM [150] (Table 3). Clinical studies of GSIs are ongoing in different malignancies.

CCL3 (MIP-1α) enhances osteoclast formation and promotes MM cell migration and survival [27]. MLN3897, a specific antagonist of the chemokine receptor CCR1, impaired osteoclastogenesis and interfered with the interactions between osteoclasts and MM cells in a preclinical setting [33] (Table 3).

## 8. Future Perspectives

The progress of new anti-myeloma drugs has contributed to excellent treatment outcomes, prolonging MM patients’ progression-free survival and overall survival, and improving their quality of life. There is still limited effective management and strong evidence regarding the restoration of bone formation or the prevention of SREs, one of the most devastating complications of MM. Furthermore, the current guidelines list the most up-to-date recommendations for MBD, but there are inconsistencies in the duration of treatment among the different guidelines. With the pathogenesis of MBD well studied and reported, emerging novel agents have recently been utilized in real-world clinical practice, including BHQ880, sotatercept, and ibrutinib. We are looking forward to positive clinical outcomes of novel agents in the future, which will offer better treatment choices for MM patients and for the prevention and therapy of MBD.

## 9. Summary

We discussed the pathogenesis of myeloma-related bone disease, the current treatment and the novel agents in development for the treatment of MBD. We also illustrated the important signaling cascades, including the RANKL/RANK/OPG axis, Notch signaling, the Wnt/β-Catenin signaling pathways, and key signaling molecules, such as DKK-1, sclerostin, osteopontin, activin A, chemokines, and interleukins, associated with osteoclast, osteoblasts, and BMSCs in the BM microenvironment. The complexity of cross relationships between MM cells and the surrounding cells has a critical role in MBD. Bisphosphonates and denosumab remain the first-line standard therapy for MM patients to prevent SREs. In development, there are new bone-modifying agents that target different molecular pathways for restoring bone metabolism; some of these are currently in clinical trials and may help meet patients’ needs in the near future.

## Figures and Tables

**Table 1:** Current myeloma bone-targeting treatment recommendations.

Guideline	Recommendations	Treatment Duration
NCCN [110]	All patients receiving primary myeloma therapy should be given bisphosphonates (category 1) or denosumab. Both pamidronate and zoledronic acid have shown equivalence in terms of reducing risk of skeletal-related events in randomized trials. Denosumab is preferred in patients with renal insufficiency.	Bisphosphonates (category 1) or denosumab for up to 2 years. Continuing beyond 2 years should be based on clinical judgment.
EHA-ESMO [113]	All patients with osteolytic disease at diagnosis should be treated with antiresorptive agents, i.e., zoledronic acid [I, A] or denosumab [I, A], in addition to specific anti-myeloma therapy.	For patients who have not achieved a PR after initial therapy, zoledronic acid should be given for more than two years. For patients who have achieved CR or VGPR, 12–24 months of therapy with zoledronic acid is adequate. At relapse, zoledronic acid has to be reinitiated. In cases of osteonecrosis of the jaw (ONJ), bisphosphonates or denosumab should be discontinued and may be re-administered if ONJ has healed.
IMWG [11]	Zoledronic acid (regardless of the presence of MBD on imaging) for patients with NDMM or RRMM; also consider for patients at biochemical relapse. Denosumab (only in the presence of MBD on imaging; also consider for patients with renal impairment).	Monthly zoledronic acid during initial therapy and in patients with less than VGPR. If patients achieve a VGPR or better after receiving monthly administration for at least 12 months, the treating physician can consider decreasing the frequency of dosing to every 3 months or, on the basis of osteoporosis recommendations, to every 6 months or yearly, or discontinuing zoledronic acid. If discontinued, it should be reinitiated at the time of biochemical relapse to reduce the risk of new bone event at clinical relapse. Continuous and monthly denosumab. If discontinued, a single dose of zoledronic acid should be given to prevent rebound effects at least 6 months after the last dose of denosumab; also consider giving denosumab every 6 months.

CR: complete response; EHA-ESMO: European Hematology Association and European Society for Medical Oncology; IMWG: International Myeloma Working Group; MBD: myeloma-related bone disease; NCCN: National Comprehensive Cancer Network; NDMM: newly diagnosed multiple myeloma; PR: partial response; RRMM: relapsed or refractory myeloma; VGPR: very good partial response.

**Table 2:** Major trials of RANKL inhibitor denosumab in myeloma bone diseases.

Trial	Study Design	Patient Numbers	Outcomes/Results	References
Phase II, open-label trial	Denosumab 120 mg SC on days 1, 8, and 15 of cycle 1 (28 days), and then day 29 (day 1 of cycle 2) and on day 1 of every cycle (28 days) thereafter	96	Suppressed bone resorption, decreased sCTx both in relapsed and plateau-phase groups, mPFS: 2.7 months (relapsed group), 8 months (plateau-phase group)	[124]
Phase III, international, double-blind, randomized, active-controlled trial	Denosumab 120 mg SC Q4W vs Zoledronic acid 4 mg IV Q4W	180	Similar time to first on-study SRE; worse OS, similar rates of overall AEs; greater suppression of uNTx	[123]
Phase III, international, double-blind, double-dummy, randomized, active-controlled trial	Denosumab 120 mg SC Q4W vs Zoledronic acid 4 mg IV Q4W	1718	Non-inferior in time to first SREs; similar incidence of ONJ; similar OS; similar time to first-and-subsequent SREs Fewer first on-study SREs (in 196 Asian patients)	[125,132]

AEs: adverse events; IV: intravenously; SC: subcutaneously; sCTx: serum C-terminal telopeptide of type I collagen; SRE: skeletal-related event; ONJ: osteonecrosis of the jaw; OS: overall survival; mPFS: median progression-free survival; uNTx: urinary N-terminal telopeptide of collagen type 1; NDMM: newly diagnosed multiple myeloma; RRMM: relapsed or refractory myeloma.

**Table 3:** Novel agents in preclinical research and clinical settings for MBD.

Drug Name	Mechanism	Therapeutic Implication	Trial Status	Results	References
BHQ880	Human neutralizing IgG1 anti-DKK1 monoclonal antibody Wnt pathway signaling	Reverse the effects of DKK1-induced osteoblast inhibition, leading to increased bone mass mediated via upregulation of osteoblasts	Phase I/II, RRMM Phase IB, RRMM	Dual therapy with zoledronic acid and BHQ880 may provide an effective treatment strategy for MBD	[143,144]
Sotatercept (ACE-011)	Decay receptor-neutralizing Activin-A, a recombinant activin receptor type IIA (ActRIIA) ligand trap	Reverse osteoblast inhibition	Phase I, RRMM Phase IIa, NDMM, RRMM	Increased hemoglobin levels (dose–response relationship), improved bone formation biomarkers	[61,146]
RAP-011	A murine ortholog of sotatercept (Activin receptor type II Murine Fc Protein)	Reverse osteoblast inhibition	Preclinical setting		[145]
Tabalumab (LY2127399)	Human IgG4 anti-BAFF monoclonal antibody	Decrease myeloma tumor burden, decrease osteoclastogenesis	Phase I, RRMM Phase II, RRMM	No PFS benefit Higher dose of 300 mg tabalumab did not improve efficacy compared to the 100 mg dose	[68,147,148]
Ibrutinib	Bruton tyrosine kinase inhibitor (BTKi)	Decrease myeloma tumor burden, decrease osteoclastogenesis	Phase II, RRMM	Clinical benefit and favorable safety/tolerability profile	[149]
Romosozumab (AMG 785)	Humanized monoclonal IgG2 anti-Sclerostin monoclonal antibody	Decreased RANKL/OPG ratio, decrease osteoclastogenesis	Preclinical setting Phase III, Osteoporosis	Decrease vertebral fracture risk in postmenopausal women with osteoporosis	[89,91]
RO4929097, γ-secretase inhibitor XII (GSI XII)	Notch/γ-secretase inhibitor Notch signaling	Downregulate CXCR4/SDF1 chemokine axis, decrease osteoclastogenesis, reduce angiogenesis	Preclinical setting		[150,151]
MLN3897	Antagonist of the chemokine receptor CCR1 Akt signaling	Inhibit CCL3-induced osteoclast formation and function	Preclinical setting		[33,152]

## Data Availability

Not applicable.
